# Supplementary material for: Controlled iris radiance in a diurnal fish looking at prey
Source: R Soc Open Sci. 2018 Feb 21;5(2):170838. doi: 10.1098/rsos.170838 (PMC5830713; doi:10.1098/rsos.170838)
Supplement: Comparison between four common reflector types (figure) [file rsos170838supp1.docx]

**Controlled ocular radiance in a diurnal fish looking at prey**

Nico K. Michiels, Victoria C. Seeburger, Nadine Kalb, Melissa G. Meadows, Nils Anthes, Amalia Mailli, Colin B. Jack

**ESM S1**

**Figure S1: Comparison between four common reflector types.** A. Focusing eyes work as a retroreflector. By focusing light from an object onto the back of the eye structure, the reflected light is sent back to the source. The brightness of the returned light depends on the reflectiveness of the layer behind the lens (e.g. a tapetum). B. Specular mirrors reflect the light at an angle that is identical to the incoming angle. They send light back to the source only when it arrives orthogonal to the mirror’s surface. Silvery fish scales have specular properties. C. Diffuse reflectors scatter incoming light in all directions. Matt structures are diffuse reflectors. D. Reflective cups show complex patterns of specular reflection, but have a higher probability to send light back to the source than a flat specular mirror. Although this may explain the strength and directionality of the reflections seen in the eyes of some invertebrates such as copepods, the actual reflective properties of copepod eyes remain to be investigated.
