# Supplementary material for: Controlled iris radiance in a diurnal fish looking at prey
Source: R Soc Open Sci. 2018 Feb 21;5(2):170838. doi: 10.1098/rsos.170838 (PMC5830713; doi:10.1098/rsos.170838)
Supplement: Species list for figure 1 (text) [file rsos170838supp2.docx]

**Controlled ocular radiance in a diurnal fish looking at prey**

Nico K. Michiels, Victoria C. Seeburger, Nadine Kalb, Melissa G. Meadows, Nils Anthes, Amalia Mailli, Colin B. Jack

**ESM S2**

List of all the species shown in figure 1 in the main text.

**No. Species Marine/freshwater**

1 *Anomalops katoptron* (Anomalopidae) Marine

2 *Bryaninops natans* (Gobiidae) Marine

3 *Hyphessobrycon pulchripinnis* (Characidae) Fresh

4 *Serrasalmus nattereri* (Characidae) Fresh

5 *Barbonymus schwanenfeldii* (Cyprinidae) Fresh

6 *Leuciscus rutilus* (Cyprinidae) Fresh

7 *Ecsenius lividanalis* (Blenniidae) Marine

8 *Heteroconger hassi* (Congridae) Marine

9 *Ctenopharyngodon idella* (Cryprinidae) Fresh

10 *Danio malabaricus* (Cyprinidae) Fresh

11 *Spicara maena* (Centracanthidae) Marine

12 *Xyrichthys novacula* (Labridae) Marine

13 *Paracheirodon axelrodi* (Characidae) Fresh

14 *Pseudotropheus socolofi* (Cichlidae) Fresh

15 *Archamia macroptera* (Apogonidae) Marine

16 *Belontia signata* (Belontiidae) Fresh

17 *Epinephelus costae* (Serranidae) Marine

18 *Serranus cabrilla* (Serranidae) Marine

19 *Sarpa salpa* (Sparidae) Marine

20 *Anthias anthias* (Serranidae) Marine

21 *Labrus mixtus* (Labridae) Marine

22 *Caesio cuning* (Caesionidae) Marine

23 *Sebastes caurinus* (Sebastidae) Marine

24 *Embiotoca lateralis* (Embiotocidae) Marine
