# Supplementary material for: Controlled iris radiance in a diurnal fish looking at prey
Source: R Soc Open Sci. 2018 Feb 21;5(2):170838. doi: 10.1098/rsos.170838 (PMC5830713; doi:10.1098/rsos.170838)
Supplement: Experimental design (2 figures, A and B) [file rsos170838supp3.docx]

**Controlled ocular radiance in a diurnal fish looking at prey**

Nico K. Michiels, Victoria C. Seeburger, Nadine Kalb, Melissa G. Meadows, Nils Anthes, Amalia Mailli, Colin B. Jack

**ESM 5 S5 A, B**

**Experimental setup and spectral properties of ocular sparks**

**
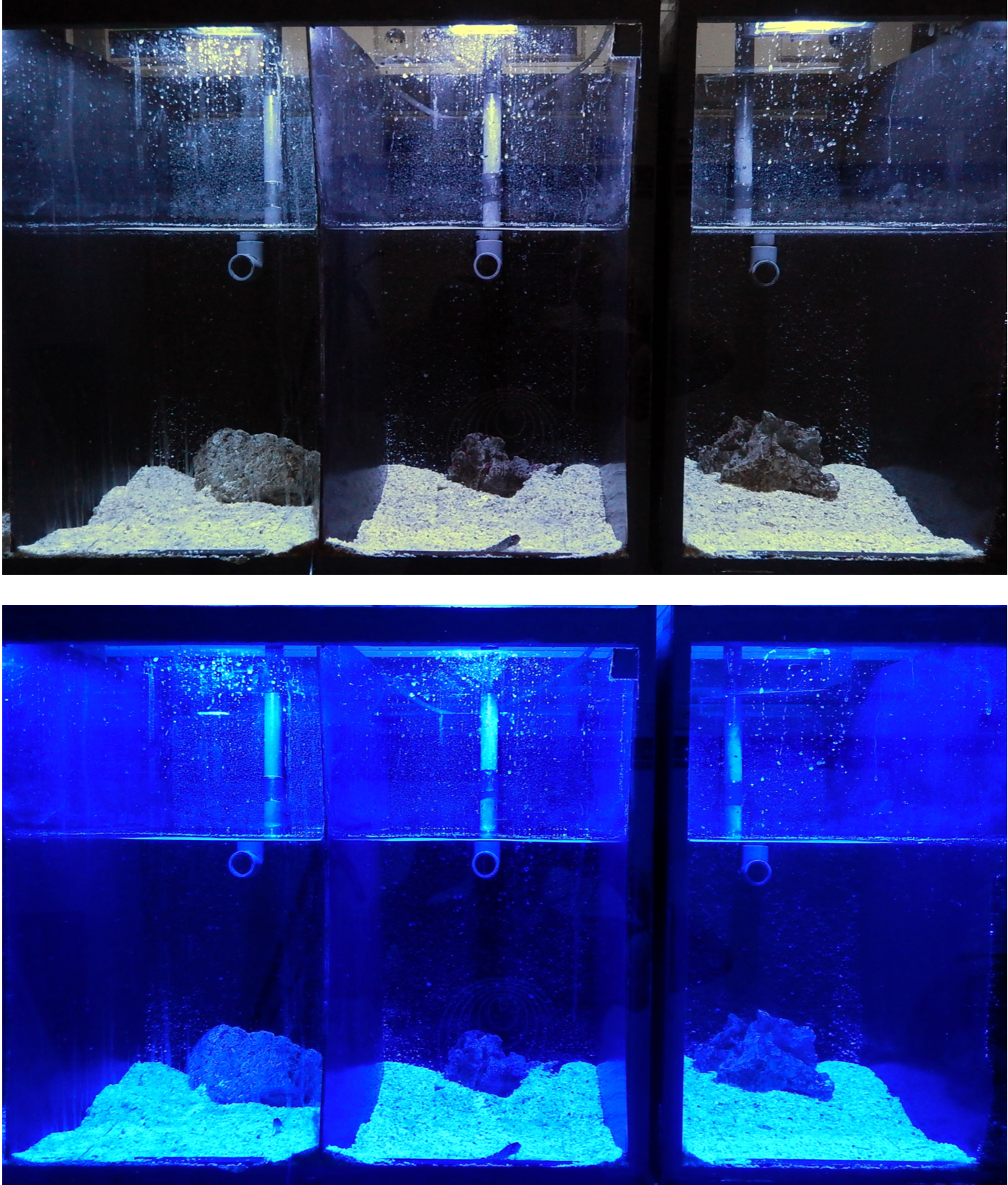
**

**Figure S5-A:** Frontal view of three of the 21 experimental tanks in the setup, each section with its own blue LED source. A *Tripterygion delaisi* individual is visible at the front window of the middle tank. The top image shows the section with manual white balance, which approximates how humans perceive the setup once adapted to the blue light. Setting the camera to automatic white balance (below) illustrates how the setup appears to a human immediately upon entering the room from a regular, broad-spectral environment. We do not know how fish perceive colour in a blue environment like this – but a certain degree of colour constancy (neural compensation for a skewed ambient spectrum), as in the top image, is expected (images taken with a Nikon AW130 by Gregor Schulte).


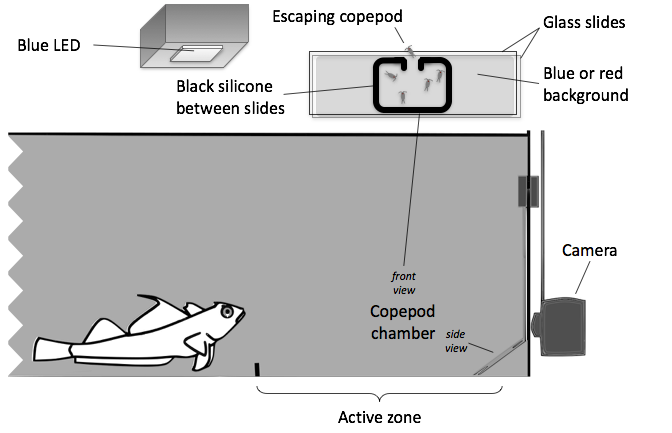


**Figure S5-B:** Side view of the experimental setup (not drawn to scale) including a copepod chamber (front view shown at the top).
