# Supplementary material for: Controlled iris radiance in a diurnal fish looking at prey
Source: R Soc Open Sci. 2018 Feb 21;5(2):170838. doi: 10.1098/rsos.170838 (PMC5830713; doi:10.1098/rsos.170838)
Supplement: Measurements of eyeshine in the copepod T. californicus (figure) [file rsos170838supp4.docx]

**Controlled ocular radiance in a diurnal fish looking at prey**

Nico K. Michiels, Victoria Seeburger, Nadine Kalb, Melissa G. Meadows, Nils Anthes, Amalia Mailli, Colin Bruce Jack

**ESM S6**

**Figure S6**: Spectral reflectance of all three ocelli of 22 live *Tigriopus californicus* copepods expressed as a proportion of the coaxial light reflected relative to a Spectralon diffuse reflectance standard (or a metal mirror top right). Top row shows the measurements of the brightest of three ocelli only, showing all curves (top left, *n* = 22 copepods), a statistical summary (top centre) and reflectance relative to a metal mirror (top right). Bottom: statistical summaries for the ocellus of intermediate (left) and lowest brightness (centre), as well as for the central red pigmented area of the eye and the cephalothorax (cuticle). Variation in ocellus brightness does not depend on ocellus identity, but on orientation of that ocellus relative to the observer. **Methods**: Live copepods were glued to the tip of a fine insect pin using Surgibond superglue and submerged in a seawater-filled diffuse white Teflon dish with the nauplius eye facing upward. The sample was observed under a Leica DM 5000B microscope using a Leica HCX APO 40x/0.80 U-V-I lens for liquid cell cultures. The microscope was modified to allow for perfect coaxial illumination using a cold light source (Schott KL 2500). Radiance measurements were taken with a PhotoResearch PR-740 radiospectrometer through the microscope. Eyeshine measurements were repeated 2-3 times per ocellus. Internal glare in the microscope accounted for less than 1% of the signal and was subsequently ignored. The graphs show that in this setup, copepod ocelli reflect up to two times as strong as a diffuse white standard and up to 50% as strong as a specular mirror. The high value of the diffuse white standard relative to the mirror can be attributed by the short (3.3 mm) working distance of the objective, allowing much of the diffusely reflected light to be captured by the nearby lens.
